# Canine Teeth with Two Roots

**Published:** 1857-07

**Authors:** 


*Canine Teeth with two Roots.*—As examples of anomalous cases of this kind are somewhat rare, the senior editor begs to acknowledge the receipt of two lower cuspids from a professional friend, each of which has two well developed roots of nearly half an inch in length. He intends placing them in the Museum of the Baltimore College of Dental Surgery, which, perhaps, already contains a larger number of anomalous and morbid specimens of teeth than is to be found in any other collection in the world.

